# Stem cell-derived extracellular vesicles as immunomodulatory agents: targeting pathological crosstalk in systemic lupus erythematosus and multiple sclerosis

**DOI:** 10.3389/fmed.2026.1796038

**Published:** 2026-05-14

**Authors:** Lifei Yao, Qiong Li, Wei Peng, Aimeng Sun, Shaofen Li, Mengting Zou, Xianyun Xu

**Affiliations:** 1 Department of Clinical Laboratory Medicine, Ji'an Central People's Hospital, Ji'an, Jiangxi, China; 2 Ji'an Central Blood Station, Ji'an, Jiangxi, China

**Keywords:** experimental autoimmune encephalomyelitis, immunomodulation, multiple sclerosis, stem cell-derived extracellular vesicles, systemic lupus erythematosus

## Abstract

Systemic lupus erythematosus (SLE) and multiple sclerosis (MS) are chronic immune-mediated diseases characterized by overlapping clinical presentations and shared immunoregulatory pathways. Both conditions involve dysregulated immune cell activation, autoantibody production, cytokine imbalance, compromised blood-brain barrier (BBB), and mechanisms that establish self-perpetuating cycles that drive neuroinflammatory cascades, demyelination, and tissue injury. Stem cell-derived extracellular vesicles (SC-EVs) efficiently deliver and protect bioactive cargo, notably key immunoregulatory molecules including microRNAs (miRNAs) and proteins, from enzymatic degradation through their bilayer membrane structure, facilitating intercellular communication and immune modulation. Preclinical studies in animal models of SLE and experimental autoimmune encephalomyelitis (EAE, the standard MS model) have demonstrated that mesenchymal stem cell-derived extracellular vesicles (MSC-EVs) modulate immune responses by suppressing pro-inflammatory mediators, enhancing anti-inflammatory signaling, promoting tissue repair, and conferring neuroprotection. Accumulating evidence suggests that the functional cargo of MSC-EVs targets key pathogenic processes in both diseases, including immune cell polarization, cytokine regulation, and tissue regeneration. This review examines the convergent immunomodulatory effects and mechanisms of SC-EVs in SLE and MS, highlighting their potential as cell-free immunotherapeutic agents for mitigating autoimmune-mediated damage. This review highlights the synergistic role of microRNA-146a-5p (miR-146a-5p) and microRNA-21-5p (miR-21-5p) in reprogramming immune responses and the context-dependent regulation of the hypoxia-inducible factor-1α (HIF-1α) axis in tissue repair.

## 1 Introduction

Systemic lupus erythematosus (SLE) and multiple sclerosis (MS) are two prototypical chronic autoimmune diseases with distinct primary targets; however, they share substantial pathogenic overlap (1, 2). SLE is a systemic disorder driven by the loss of immune tolerance, autoantibody production, and multi-organ inflammation (1). In contrast, MS primarily affects the central nervous system (CNS), where immune-mediated blood-brain barrier (BBB) disruption, demyelination, and axonal injury cause progressive neurological deficits (2). Both diseases share core immunopathological features, including dysregulated self-reactive T and B cells, persistent inflammatory amplification, and impaired barrier integrity at the tissue interfaces (1, 2).

Although their principal targets differ, SLE and MS share several pathogenic features, including dysregulated T- and B-cell responses, persistent inflammatory amplification, and impaired barrier integrity (3, 4). In SLE, inflammatory involvement may extend to the CNS and cause neuropsychiatric manifestations that closely mimic MS, particularly when demyelinating features are present (5–8). These shared symptoms include cognitive impairment, fatigue, and sensory disturbances, complicating the diagnosis, particularly in cases with indolent progression or incomplete clinical pictures (6–8). The most significant pathological overlap between these two diseases occurs at tissue interfaces and involves processes that regulate vascular and BBB integrity, immune cell trafficking, glial-immune crosstalk, and focal inflammation (9, 10). Given the differing treatment strategies for these two conditions, diagnostic confusion may compromise their efficacy and safety (11–13). In these scenarios, therapeutic strategies may shift toward broad-spectrum immunomodulators that address shared immune dysfunction across tissues and barrier interfaces rather than disease-specific downstream interventions dependent on a definitive diagnosis.

Remodeling of the immune microenvironment is central to both diseases. During chronic inflammation, immune cells release bioactive factors through autocrine and paracrine mechanisms to regulate responses in local and distant tissues (14). Among the mediators of intercellular communication, extracellular vesicles (EVs) have emerged as critical signaling carriers between immune cells and target tissues (15, 16). The term “extracellular vesicles” is now generally adopted (17) for these nano- to micrometer-sized membrane-bound vesicles that are actively secreted by cells into the extracellular environment (18). EVs are a heterogeneous population of particles that are broadly categorized by their biogenesis into exosomes (endosomal origin from multivesicular bodies), microvesicles (shed via budding from the plasma membrane), and apoptotic bodies (19, 20). By carrying diverse bioactive cargoes, including proteins, lipids, and nucleic acids, and protecting them from degradation within a lipid bilayer, EVs mediate local and long-distance intercellular signaling and contribute to immune regulation, inflammatory responses, and tissue repair (15, 16, 19, 21).

In recent years, stem cell-derived extracellular vesicles (SC-EVs), particularly mesenchymal stem cell-derived extracellular vesicles (MSC-EVs), have attracted attention as cell-free therapeutic candidates (22, 23). MSC-EVs actively modulate immune responses rather than merely reflect disease states by inheriting the profound immunomodulatory and regenerative properties of their parent stem cells, while circumventing many practical and safety limitations associated with direct cell transplantation (22, 23). Preclinical studies suggest that MSC-EVs can attenuate systemic immune dysregulation in SLE and reduce neuroinflammation and demyelination in MS (24, 25).

Whether EVs-mediated pathways converge on common immunoregulatory mechanisms in both diseases and whether this convergence can inform shared therapeutic targets remains an open question. Therefore, this review aims to provide an integrative framework for understanding the overlap between SLE and MS. Specifically, we summarized their shared immunopathological mechanisms, identified the miR-146a-5p/miR-21-5p/hypoxia-inducible factor-1α (HIF-1α) axis as a cross-disease regulatory node, and discussed how SC-EVs, particularly MSC-EVs, could be leveraged as a cell-free therapeutic strategy.

## 2 Clinical overlap between SLE and MS

Although SLE and MS are distinct autoimmune entities, extensive documentation indicates partial clinical overlap (6–8, 26, 27). Retrospective studies and case reports have demonstrated that patients with SLE frequently develop neurological manifestations characterized by CNS demyelination, which is clinically and radiologically indistinguishable from MS (6–8, 26–28). SLE and MS share striking similarities in terms of age of onset, sex distribution, and disease course, predominantly affecting young to middle-aged women and frequently following a relapsing-remitting pattern (7, 29). Non-specific features such as fatigue, spinal cord involvement, and diffuse white matter lesions on brain magnetic resonance imaging (MRI) further reduce clinical discriminability (7, 29). While MS primarily targets the CNS, neuropsychiatric SLE (NPSLE) can also cause seizures, headaches, cognitive impairment, cerebrovascular events, and psychiatric symptoms (7, 29). Cognitive dysfunction is a hallmark of both conditions and substantially impairs quality of life (7, 29, 30).

Although both disorders have relatively well-established diagnostic criteria (31, 32), their early presentations are often non-specific. Approximately 39%−50% of patients with SLE present with neurological symptoms as their initial manifestation (33), while multifocal CNS lesions in MS may produce a clinical picture resembling multisystem disease. Imaging and cerebrospinal fluid (CSF) analyses provide supportive evidence, but not definitive evidence. Neuropsychiatric SLE often shows relatively static small-vessel lesions or changes associated with dementia on MRI, including subcortical and periventricular white matter T2 hyperintensity, brain atrophy, ischemia, hemorrhage, and vasculitis (34–37). MS typically presents with oval periventricular lesions, corpus callosum, and spinal cord involvement, and dynamic disease progression. CSF oligoclonal bands are positive in up to 98% of MS, but only in 15%−50% of SLE (38, 39). This clinical and radiological overlap increases the risk of misdiagnosis and may lead to inappropriate treatment and potential iatrogenic harm. While true comorbidity of SLE and MS is relatively uncommon, MS-like CNS demyelinating phenotypes in patients with SLE are frequently encountered in clinical practice. The two diseases differ substantially in terms of treatment approaches and drug safety profiles (12, 13, 40). MS management centers on disease-modifying therapies targeting neuroinflammation, including interferon β, sphingosine-1-phosphate receptor modulators, fumarate esters, and monoclonal antibodies (12, 13). SLE treatment relies primarily on systemic immunosuppression and immunomodulation, including hydroxychloroquine, glucocorticoids, antimetabolites, and B cell-targeted therapies (40). Certain medications commonly used for MS can induce or exacerbate SLE, whereas several drugs used for systemic autoimmune diseases may worsen or trigger CNS demyelination (6). Interferon-β (IFN-β) has been reported to cause and aggravate SLE. Although tumor necrosis factor-α (TNF-α) inhibitors are widely used to treat systemic autoimmune diseases, they are contraindicated in MS because of their potential to induce demyelination (12, 41). Conversely, certain immunosuppressants commonly used in SLE, such as mycophenolate mofetil, methotrexate, and anti-CD20 therapy, are generally considered safe and may be beneficial for patients with overlapping symptoms. However, their use in MS is largely off-label (42). Given the potential for diagnostic uncertainty and treatment conflicts, identifying shared and modifiable immunoregulatory targets offers significant translational value for patients with overlapping phenotypes.

## 3 Shared pathophysiological alterations in SLE and MS

**Figure 1:**
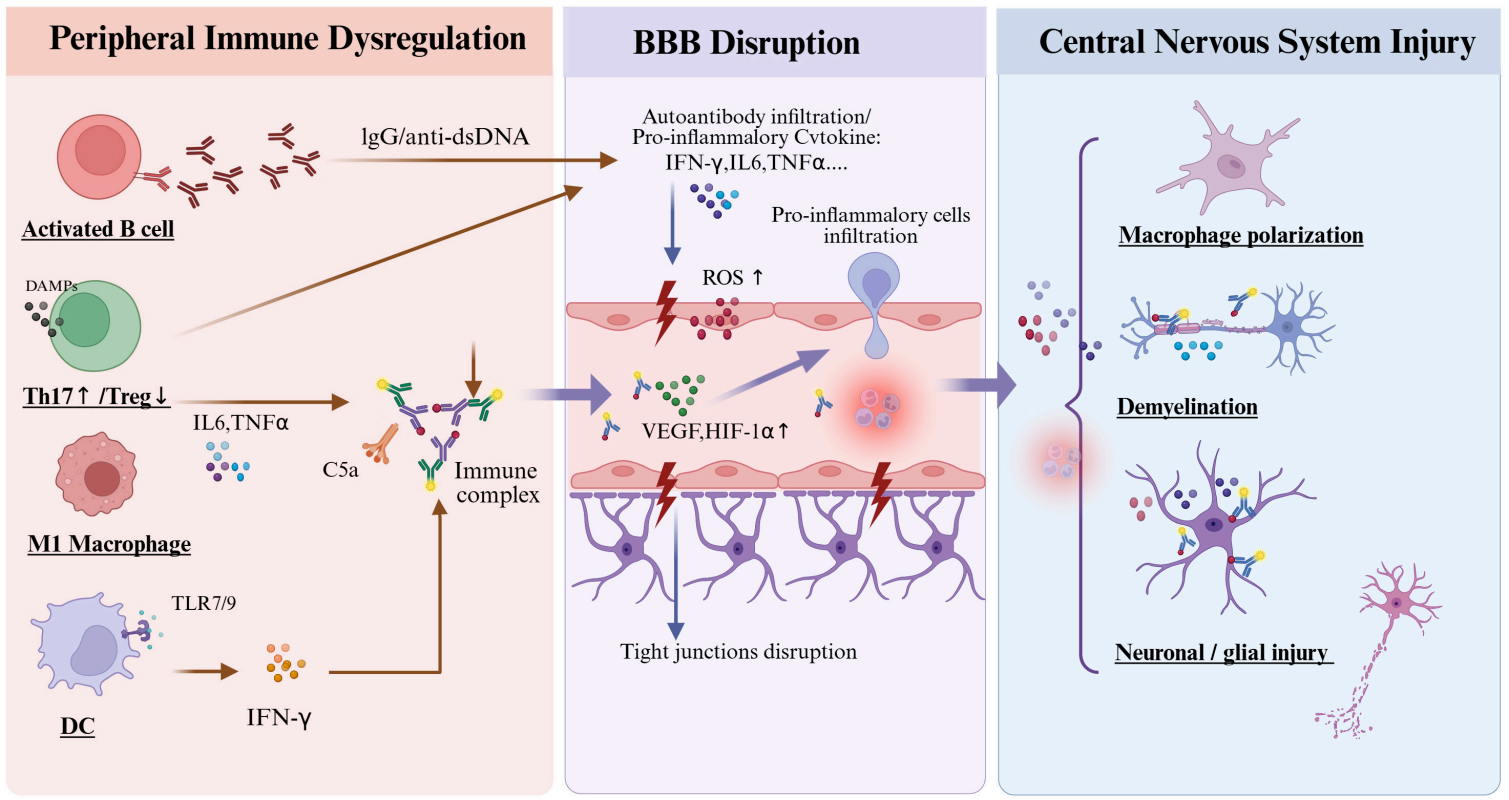
Convergent pathological cascade from peripheral immune dysregulation to BBB disruption and CNS injury in SLE or EAE. In the periphery, activated B cells, Th17/Treg imbalance, pro-inflammatory M1 macrophage polarization, and DC activation drive autoantibody production and immune-complex formation. These events trigger systemic cytokine release and complement activation, leading to endothelial stress and increased BBB permeability. At the neurovascular interface, autoantibody infiltration and ROS, alongside upregulated VEGF and HIF-1α, disrupt tight junction integrity. Subsequent leukocyte infiltration into the CNS fuels microglial activation, neuroinflammation, and demyelination, ultimately leading to neuronal and glial injury. SLE, systemic lupus erythematosus; EAE, experimental autoimmune encephalomyelitis; BBB, blood–brain barrier; CNS, central nervous system; dsDNA, double-stranded DNA; HIF-1α, hypoxia-inducible factor-1 alpha; IFN-γ, interferon-gamma; IL, interleukin; DC, dendritic cell; ROS, reactive oxygen species; SLE, systemic lupus erythematosus; MS, multiple sclerosis; Th17, T helper 17 cell; TNF-α, tumor necrosis factor-alpha; Treg, regulatory T cell; VEGF, vascular endothelial growth factor; TLR9, Toll-like receptor 9; TLR7, Toll-like receptor 7; C5a, complement component 5a; DAMPs, damage-associated molecular patterns.

SLE and MS share substantial pathophysiological commonalities, primarily manifested as abnormal immune cell activation, dysregulated cytokine networks, and impaired BBB function (43, 44). These alterations collectively remodel the CNS microenvironment, inducing focal inflammation and hypoxic conditions that drive neuroinflammation and demyelination in susceptible individuals (43, 44). As illustrated in Figure 1, understanding these convergent mechanisms is vital for developing targeted interventions, particularly through SC-EVs in preclinical models, such as experimental autoimmune encephalomyelitis (EAE) and lupus-prone mice, in which EVs can modulate these shared pathways to interrupt pathological crosstalk.

### 3.1 Immune cell polarization

Immune responses in SLE and MS depend on innate immune recognition of tissue injury signals that coordinate adaptive immunity (45). The innate immune system acts as the first line of defense, maintaining immune-inflammatory homeostasis, whereas adaptive immunity provides antigen-specific responses with a memory capacity (45). However, pathological polarization drives a transition in adaptive immunity, leading to a persistent inflammatory cascade resistant to self-limitation, ultimately causing chronic tissue damage (46). In MS, damaged CNS tissue releases damage-associated molecular patterns (DAMPs), such as nucleoproteins and nucleic acids, which trigger immune activation in MS (47). These DAMPs bind to pattern recognition receptors (PRRs), activating macrophages and microglia, and disrupting local homeostasis (48). Specifically, this activation predominantly drives microglia and infiltrating macrophages toward a pro-inflammatory M1 phenotype, with a corresponding increase in pro-inflammatory cytokines and immune markers detectable in the CSF, further exacerbating demyelination and tissue injury (49). M1-polarized microglia/macrophages secrete neurotoxic cytokines and reactive oxygen species, further exacerbating demyelination and tissue injury (49). Conversely, the transition to an anti-inflammatory M2 phenotype is essential for tissue repair, remyelination, and the resolution of neuroinflammation. However, this process is often impaired in chronic MS lesions (50). This process leads to aberrant antigen presentation, promoting T-cell differentiation toward pathogenic T helper 1 (Th1) and T helper 17 (Th17) phenotypes, while impairing regulatory mechanisms (51). A similar cascade occurs in SLE but with more systemic effects (46). Immune complexes formed by endogenous nucleic acids and autoantibodies persistently stimulate PRR signaling pathways, particularly in dendritic and B cells, thereby sustaining chronic inflammation. B cells drive autoantibody production and reinforce pathological T cell polarization through antigen presentation and co-stimulatory signals, exacerbating the breakdown of immune tolerance (52). Furthermore, systemic inflammation in SLE is heavily reliant on aberrant macrophage polarization (53). Circulating and tissue-resident macrophages predominantly skew toward the classical M1 phenotype, contributing to the massive production of pro-inflammatory cytokines and defective efferocytosis (24). Meanwhile, reparative M2 polarization is significantly suppressed, accelerating multi-organ damage, such as lupus nephritis and alveolar hemorrhage (53). In both diseases, sustained innate immune activation reshapes adaptive cell polarization, creating positive feedback loops that prevent inflammation resolution and extend autoimmune damage from local to systemic levels (46, 52). These polarization dynamics have been recapitulated in preclinical EAE and lupus models, where MSC-EVs have been shown to convert pro-inflammatory macrophages/microglia toward anti-inflammatory phenotypes and restore the Th17/Treg balance (24, 50, 54–57).

### 3.2 Adaptive immune dysregulation

Following the initial priming of innate immunity, profound dysregulation of adaptive immune networks sustains chronicity in both MS and SLE (46, 51). The architectural hallmark of this shared pathology is the collapse of CD4^+^ T-cell homeostasis, defined by a skewed Th17/Treg (51, 58). In MS, driven by IL-6 and TGF-β, the dominant STAT3 transcriptional program enhances RORγt-dependent Th17 effector states while suppressing Foxp3-mediated immune tolerance (59). This triggers the release of a cascade of IL-17A, IL-21, and granulocyte-macrophage colony-stimulating factor (GM-CSF), leading to leukocyte infiltration, endothelial dysfunction, and tissue injury (59).

This pathogenic axis is reinforced by sustained T–B cell co-stimulation, type I interferon-driven plasmablast expansion, and profound metabolic reprogramming via mechanistic target of rapamycin (mTOR) (59). Although MS and SLE share similar upstream immune regulatory defects, their terminal effector pathways are different. The adaptive immune response in MS is highly compartmentalized. After crossing the BBB, autoreactive Th1 and Th17 cells are locally reactivated, mediated by microglia and infiltrating antigen-presenting cells, leading to focal demyelination and axonal injury (49, 51). In contrast, SLE primarily manifests as dysregulation of the systemic humoral immune system. Abnormal B-cell activating factor (BAFF) signaling and excessive endosomal TLR activation persistently drive plasma cell proliferation and autoantibody production (46, 52).

Consequently, neurologic injury in neuropsychiatric SLE represents a distinct paradigm, emerging from a systemic breach of autoantibodies and complement into a permissive neurovascular niche, driving diffuse gliosis and microvascular degradation (30, 60).

### 3.3 Cytokines in inflammatory response

The central paradox of neuroimmunology is that fundamentally different autoimmune triggers can lead to strikingly similar patterns of CNS damage. SLE and MS exemplify this. Despite their distinct initiating cytokine cascades, both ultimately breach the BBB and drive demyelination through convergent inflammatory pathways (43, 44, 51).

The pathogenesis of SLE is primarily driven by systemic interferon-driven humoral immune disruption. Apoptotic nucleic acids activate TLR7/9 on plasmacytoid dendritic cells, initiating an IRF7/STAT1-dependent type I interferon amplification loop that promotes BAFF-dependent activation of autoreactive B cells, leading to immune complex formation and complement-mediated injury of the cerebral microvasculature (46, 61). Additionally, IL-21 produced by T follicular helper (Tfh) cells activates the STAT5/BCL6 signaling pathway in extrafollicular B cells, exacerbating B-cell dysfunction and lupus nephritis (62). In contrast, the pathogenesis of MS arises from focal T-cell-centric attacks. IL-23 serves as a primary driver, promoting STAT3/RORγt-dependent Th17 cell polarization and CCR6-mediated migration to the CNS, where these cells secrete IL-17 (63). IL-17 induces local MMP-9 expression, disrupting endothelial tight junctions and compromising the BBB, thereby paving the way for parenchymal infiltration and targeted oligodendrocyte destruction (64). Concurrently, IL-12 provides a second signal, biasing T cells toward a Th1 phenotype via the STAT4/T-bet pathway and upregulating CXCR3 to promote CNS infiltration (51, 65). In this scenario, demyelination primarily stems from direct T cell-mediated cytotoxicity against oligodendrocytes rather than systemic autoantibody deposition.

Following neurovascular disruption, distinct initial cascades of these diseases converge into a shared inflammatory amplification phase, which is regulated by IL-6, TNF-α, and IFN-γ (60, 61). IL-6 drives Th17 cell differentiation via the JAK/STAT3 signaling pathway while suppressing regulatory T cell (Treg) programming (61). In SLE, it also supports Tfh cell differentiation and germinal center activity. TNF-α upregulates endothelial cell adhesion molecules (ICAM-1 and VCAM-1) to facilitate leukocyte extravasation across the BBB (66). Although it's downstream effects exhibit disease specificity. In SLE, it maintains the survival and interferon production of plasmacytoid dendritic cells, whereas in MS, it damages oligodendrocytes by activating the TNFR1-p38 MAPK pathway (61, 67).

Furthermore, although IFN-γ enhances antigen presentation in both diseases, it operates in starkly different immune microenvironments. In SLE, IFN-γ synergizes with type I interferons to sustain anti-dsDNA antibody production (68). Conversely, in MS, IFN-γ induces inducible nitric oxide synthase (iNOS) expression and M1 polarization in microglia, thereby promoting oligodendrocyte apoptosis (69). Thus, the clinical overlap between MS and SLE appears to become more evident during downstream inflammatory amplification rather than during the earliest disease-initiating events. At this stage, IL-6 persistently undermines Treg-mediated immunosuppression, whereas TNF-α and IFN-γ jointly promote leukocyte extravasation and sustain a local cytotoxic state (70, 71).

The chronic progression of both diseases is aggravated by the failure of immune homeostasis and endogenous tissue repair programs. In SLE, despite compensatory increases in IL-10, defective regulatory T-cell function and dysregulated IL-10 signaling may impair negative feedback control of IL-12- and IFN-γ-driven inflammation (72). Similar defects in MS immune regulation sustain the hyperactivity of Th1 and Th17 cells (70, 73). Concurrently, TGF-β signaling is disrupted in both diseases through distinct mechanisms: impaired Smad3 nuclear translocation in SLE compromises B-cell tolerance (74), whereas reduced Smad2/3 phosphorylation in MS dysregulates Treg function, promoting pro-inflammatory Th17 responses (59, 75).

Ultimately, although SLE originates as an interferon-driven humoral immune disorder and MS arises from focal T-cell-mediated neuroinflammation, both converge on the same IL-6/TNF-α/IFN-γ inflammatory axis (60, 61). This shared terminal pathophysiology provides a compelling mechanistic rationale for the use of broad-spectrum immunotherapeutic interventions for both conditions.

### 3.4 Oxidative stress in immune activation

SLE and MS share overlapping immune-mediated pathologies, in which immune activation and BBB dysfunction act synergistically to drive disease progression rather than as independent events (76). In this context, reactive oxygen species (ROS) generation is increasingly recognized as a convergent downstream mechanism linking immune activation, vascular dysfunction, and metabolic disturbances (76, 77). Excessive ROS accumulation, resulting from an imbalance between ROS production and antioxidant capacity, leads to oxidative stress and subsequent cellular injury, including lipid, protein, and DNA damage (77). ROS typically originate from the partial reduction of oxygen molecules, with unpaired electrons in their outer shell conferring high reactivity. When ROS production exceeds the scavenging capacity of antioxidant systems, oxidative stress develops, leading to cellular dysfunction via pathways such as lipid peroxidation, protein oxidation, and DNA damage (77). Oxidative stress is closely associated with altered immune activation thresholds and sustained inflammatory responses in patients with SLE. Hypoxia-inducible factor-1α (HIF-1α) signaling is consistently upregulated in multiple autoimmune inflammatory conditions, where it modulates immune cell metabolism and effector function, thereby influencing disease activity (78).

Under neuroinflammatory and demyelinating conditions, ROS exert more direct pathogenic effects. EAE has demonstrated that excessive ROS contribute to myelin damage, glial activation, and inflammatory cell infiltration (79). Specifically, ROS generation via microglial NADPH oxidase (Nox2) correlates with disease severity and the extent of demyelination, whereas Nox2 inhibition significantly attenuates neuroinflammation (80). Crucially, at the neurovascular interface, NADPH oxidase–derived ROS further compromise BBB integrity by upregulating matrix metalloproteinase activity and degrading endothelial tight junctions, as shown in EAE models (64, 81).

### 3.5 Blood-brain barrier disruption

A defining pathophysiological feature shared by MS and SLE is the compromise of the BBB, driven by immune cell infiltration and complement activation (17, 60). This loss of integrity is mechanically pivotal, establishing a bidirectional gateway that reinforces pathological crosstalk between the periphery and CNS (17, 60). BBB breakdown facilitates the influx of immune cells into the CNS parenchyma, instigating neuroinflammation and progressive neurodegeneration, while simultaneously allowing the systemic dissemination of CNS-derived inflammatory mediators (17, 60). Moreover, immune-mediated disruption of the BBB promotes the systemic dissemination of inflammatory mediators, perpetuating widespread inflammatory cascades throughout the body (17, 60). The BBB comprises specialized capillary endothelial cells that are interconnected by tight junctions (82, 83). These cells function within the neurovascular unit alongside the basement membrane, pericytes, microglia, and astrocytes to maintain a highly selective barrier (82, 83).

In MS, disease progression is closely correlated with the extent of BBB disruption (84). Lesion evolution is associated with neovascularization and vasogenic cerebral edema, a key complication in which compromised integrity permits the extravasation of fluid, ions, and plasma proteins, thereby increasing intracranial pressure (17, 85, 86). Activated peripheral lymphocytes penetrate the barrier, initiating local immune responses that further impair endothelial function via proteolytic enzymes and inflammatory cytokines (65). These infiltrating cells secrete factors, particularly vascular endothelial growth factor (VEGF), which exacerbate permeability, support metabolic demands, and accelerate disease progression (85, 87). Furthermore, barrier breakdown enables macrophage entry into the parenchyma, amplifying tissue injury (17). HIF-1α is considered a more direct driver of BBB disruption in EAE model. During disease activity, significantly elevated HIF-1α expression synergistically weakens the structural integrity of the BBB by upregulating VEGF signaling, downregulating tight junction proteins, and enhancing astrocytic inflammatory responses. This facilitates sustained immune cell infiltration into the CNS and exacerbates local tissue injury (88).

In SLE, BBB disruption underlies diffuse neuropsychiatric manifestations and is driven by thrombo-inflammatory vascular injury, brain-reactive autoantibodies, and complement activation (30, 60). The complement component C5a triggers endothelial cell activation and cytoskeletal rearrangement via the C5a receptor (C5aR), and blocking this pathway alleviates barrier dysfunction (89). In NPSLE, anti-neuronal antibodies cross the BBB to target hippocampal neurons, driving hypermetabolism and atrophy (90). This breach further facilitates the extravasation of plasma proteins and autoantibodies, precipitating neuroinflammation (30), whereas severe barrier disruption may lead to cerebral edema and intracranial hypertension (91, 92). Under inflammatory conditions, endothelial cells upregulate adhesion molecules such as intercellular adhesion molecule-1 (ICAM-1) and vascular cell adhesion molecule-1 (VCAM-1), facilitating leukocyte transmigration into the CNS (66). Although BBB dysfunction generally portends adverse outcomes in MS and SLE, it may paradoxically enhance therapeutic drug delivery to CNS targets (16). Overall, in SLE, particularly in neuropsychiatric SLE, direct evidence for HIF-1α's role in BBB disruption remains limited, with its effects being largely indirect and context-dependent. Existing research strongly supports HIF-1α as an auxiliary factor that weakens BBB stability in chronic inflammatory and hypoxic settings by amplifying inflammatory responses and metabolic reprogramming, rather than acting as a direct driver (93).

Critically, BBB dysfunction in MS and SLE should be conceptualized not merely as a downstream consequence, but as a modifiable driver of pathological crosstalk. Therapeutic strategies focused on barrier repair or endothelial immunomodulation have the potential to restrict immune cell entry, dampen microglial activation, and limit compartmentalized inflammation.

### 3.6 Mechanisms of tissue damage

Clinical and pathological findings indicate that CNS lesions in SLE and MS converge on the common endpoints of tissue injury: neuroinflammation, demyelination, and BBB disruption (49, 94, 95). These processes are driven by immune effector mechanisms that directly target myelin or glial/neuronal support cells, whereas innate immune activation further intensifies inflammatory demyelinating lesions (49, 94, 95).

In SLE, tissue damage stems from sustained autoimmune activation following immune tolerance breakdown, which is driven by complement-mediated endothelial injury (30, 60). This vascular insult promotes leukocyte adhesion, platelet activation, and coagulation, establishing a self-reinforcing thrombo-inflammatory cycle. Consequently, compromised BBB integrity and abnormal cerebral perfusion expose the CNS to peripheral immune mediators (30, 60). Within the CNS, injury propagates through converging pathways: parenchymal inflammation mediated by complement activation, glial reactivity, cytokine signaling, and vascular pathology characterized by microvascular lesions, micro-thrombosis, and hypoperfusion (30, 60). In cases involving specific autoantibodies, such as AQP4-IgG, complement-dependent astrocytic injury further amplifies inflammation and exacerbates demyelination (96).

## 4 Immunomodulatory effects of extracellular vesicles

MSC-EVs exhibit broad immunomodulatory activity in preclinical models of SLE and EAE. Rather than targeting a single inflammatory mediator or depleting specific immune cell subsets, MSC-EVs orchestrate multiple upstream regulatory nodes in the immune network. DCs, myeloid cells, T cells, and B cells undergo functional reprogramming upon EV exposure, driving the inflammatory environment toward immune homeostasis. Although the biological consequences of these diseases differ owing to tissue-specific variations, the underlying regulatory principles remain consistent. In SLE, MSC-EVs primarily restore systemic immune tolerance, whereas in EAE models, they attenuate neuroinflammation and foster a CNS microenvironment conducive to repair (50, 97).

Sustained activation of autoreactive T cells relies on antigen presentation and co-stimulatory signals from DCs. EVs exposure disrupts this process by downregulating surface co-stimulatory molecules (CD80 and CD86) and shifting the cytokine profile (98, 99). Consequently, DCs have a reduced ability to support sustained inflammatory T-cell activation. Furthermore, EVs cargo impairs DC migration. In both SLE and EAE models, macrophages and microglia typically exhibit a pro-inflammatory M1 phenotype, characterized by impaired phagocytosis and elevated secretion of TNF-α, IL-1β, and IL-6 (100, 101). Treatment with MSC-EVs repolarizes these myeloid cells toward the M2 phenotype, which is marked by increased CD206 expression, enhanced clearance of apoptotic debris, and elevated IL-10 and TGF-β production (50, 57). This repolarization is largely driven by EV-carried regulatory miRNAs. For instance, miR-21-5p and miR-16 regulate phosphatase and tensin homolog (PTEN) and programmed cell death 4 (PDCD4) to alleviate metabolic constraints on M2 polarization (24).

As inflammatory signaling from innate immune cells wanes, the adaptive immune response undergoes a significant reconfiguration. A hallmark of both SLE and EAE is the disruption of the Th17/Treg balance (58, 102). The EV-mediated reduction in IL-6, IL-12, and TNF-α, combined with elevated TGF-β, skews CD4^+^ T cell differentiation away from the Th17 lineage and toward Foxp3^+^ Treg cells (97). EV-associated miRNAs directly suppress the pathways critical for Th17 maintenance. Pathological B cell activation in SLE, which relies on T cell and myeloid support, is similarly constrained (97, 103). EV-delivered miR-21-5p modulates the phosphatidylinositol 3-kinase/protein kinase B (PI3K-Akt) pathway to promote regulatory B cell differentiation over antibody-secreting plasma cells, whereas the miR-155/SHIP-1 axis raises the B cell activation threshold (97, 103, 104).

Although MSC-EVs engage conserved immunoregulatory pathways, their physiological impact is disease-specific. In SLE models (e.g., MRL/lpr), this manifests as systemic immune reconstitution, restored tolerance, improved B cell homeostasis, and enhanced autoantigen clearance (97). Conversely, in the EAE model, the protective effects were largely confined to the CNS (50). Suppression of neuroinflammation and establishment of an M2-dominant microglial niche support oligodendrocyte progenitor cell survival and subsequent remyelination.

## 5 The therapeutic modulatory effects of SC-EVs in EAE

MS is a human-specific disease, and existing animal models can only recapitulate certain aspects of its pathologic features. Among these, EAE is the most widely used model for evaluating the therapeutic efficacy of EVs. MSC-EVs and related stem cell populations have emerged as promising therapeutic agents for the modulation of neuroinflammation in CNS disorders. Seventeen studies conducted between 2018 and 2025 investigated MSC- or SC-EVs in various neuroinflammatory models. Most investigations (*n* = 13) utilized experimental EAE models (25, 54, 55, 105–114). Additional studies have examined virus-induced encephalomyelitis (*n* = 1) (115), lymph node-mediated inflammation (*n* = 1) (116), and *in vitro* CNS cell culture systems (*n* = 2) (50, 99). Experimental approaches predominantly employed murine models, followed by rat models, with complementary *in vitro* validation using isolated microglia, splenocytes, and T cell cultures.

Collective evidence demonstrates the robust dual immunomodulatory and neuroprotective properties of MSC-EVs. These vesicles effectively inhibited pro-inflammatory cytokine production, T cell proliferation, and CNS immune cell infiltration, consequently attenuating demyelination and improving clinical disability scores. Concurrently, MSC-EVs enhanced the expression of anti-inflammatory cytokines, promoted beneficial M2 microglial polarization, augmented regulatory T cell responses, and supported oligodendrocyte viability and myelin regeneration in the CNS. Mechanistic investigations have revealed that MSC-EVs operate through the STAT3 signaling pathway and miRNA-mediated regulation, particularly the miR-181a-5p/USP15-RelA/NEK7 axis, to prevent microglial pyroptosis and restore CNS immune homeostasis (111). Despite the variability in the cellular origins of EVs (bone marrow-, adipose tissue-, and umbilical cord-derived), therapeutic dosing protocols, and delivery routes, these convergent findings provide compelling evidence that MSC-derived EVs are effective modulators of CNS inflammation, with substantial therapeutic potential in MS and related demyelinating disorders. The comprehensive study methodologies and outcomes are listed in Table 1.

**Table 1:** Preclinical and clinical studies assessing the effect of EVs from MSCs on the regulation of MS.

References	Country	Resources	Species	Models	Targets	Main functions
Laso-García et al. (115)	Spain	ADSCs	Mice	TMEV	GFAP, Iba-1	Increase cell proliferation in the subventricular zone and decrease inflammatory infiltrates in the spinal cord
Li et al. (50)	China	BMSCs	Rats, Cells	EAE, microglia cell line model	IL-10, IL-12, TGF-β, Arg-1	Attenuate inflammation and demyelination by regulating the polarization of microglia
Clark et al. (25)	USA	PMSCs	Mice, Cells	EAE	MOG, Enpp6, MAG	Reduce DNA damage in oligodendroglia populations and increased myelination within the spinal cord
Jafarinia et al. (105)	Iran	hADSCs	Mice	EAE	MOG	Diminishes the proliferative potency of T cells, the mean clinical score, leukocyte infiltration, and demyelination.
Fathollahi et al. (106)	Iran	ADSCs	Mice, Cells	EAE	TGF-β, IFN-γ, IL-17A, IL-10	Inhibit the proliferation of autoreactive lymphocytes, promote the production of anti-inflammatory cytokines.
Ahmadvand Koohsari et al. (107)	Iran	hUMSCs	Mice	EAE	TNF-α, IFN-γ, IL-17A, IL-10	Reduce pro-inflammatory cytokines, increase anti-inflammatory cytokines, and decrease leukocyte infiltration.
Xiao et al. (108)	China	NSCs	Mice, Cells	EAE	NA	Enhance oligodendrocyte survival, inhibited myelin damage, and promote myelin regeneration as well as neuroinflammation
Wang et al. (54)	China	hUMSCs	Mice, Cells	EAE	TNF-α, IFN-γ, IL-17A, IL-10, IL-2, IL-6, IL-4	Reduce active-microglia proportions and promoted M1 to M2 microglial cell polarization through TNF pathway
Mohammadzadeh et al. (109)	Iran	hUMSCs	Mice	EAE	TGF-β, IFN-γ, IL-17, STAT3	Anti-inflammatory agent to regulate T-cells responses
Turano et al. (116)	Italy	ASCs	Mice	EAE	NA	Target in flamed lymph nodes
Manni et al. (110)	Italy	HAFSCs	Mice, Cells	EAE	IL-6, IL-11, IL-23, TNF-α	Polarize T cells toward a regulatory phenotype, restore immune homeostasis
Park et al. (55)	Korea	cAT-MSCs	Mice	EAE	STAT3	Reduce inflammatory cell infiltration and demyelination, elevate M2 macrophages and regulatory T cells via HIF-1α/STAT3 activation.
Shi et al. (111)	China	MSCs	Mice	EAE	miR-181a-5p	Inhibit microglial inflammation and pyroptosis through the USP15-mediated RelA/NEK7 axis
Vakili et al. (112)	Iran	BMSCs	Mice	EAE	TNF-α, TGF-β, IL-1β, IL-6, IL-10	Reduce splenocyte proliferation, increase T cell frequency, shift cytokine profiles toward reduced pro-inflammatory and increased anti-inflammatory cytokines
Shahryari et al. (113)	Iran	MSCs	Mice, Cells	EAE	miR-146a-5p (enriched EVs)	Immunomodulatory and neuroprotective effects; suppress NF-κB pathway and modulate HIF-1α-related neuroinflammation, reduce neuroinflammation, and promote myelin preservation
Reis et al. (99)	United Kingdom	MSCs	Cells	Dendritic cells (*in vitro*)	miR-21-5p	Attenuate dendritic cell maturation and function; block DC migration via CCR7 targeting, promote tolerogenic state
Wu et al. (114)	China	BMSCs	Mice	EAE	IFN-γ, IL-17A, IL-4, IL-10	Reduce inflammatory infiltration and demyelination by promoting the expression of tight junction (TJ)-related markers in bEnd3 cells

EVs, extracellular vesicles; BMSCs, bone marrow mesenchymal stem cells; EAE, experimental autoimmune encephalomyelitis; IL, interleukin; TGF, transforming growth factor; IFN, interferon; TNF, tumor necrosis factor; Arg1, arginase-1; ADM/SCs, adipose tissue-derived mesenchymal stem cells; TMEV, Theiler's murine encephalomyelitis virus; PMSCs, placenta-derived MSCs; MOG, myelin oligodendrocyte glycoprotein; Enpp6, ectonucleotide pyrophosphatase/phosphodiesterase 6; MAG, myelin associated glycoprotein; hADSCs, human adipose-derived mesenchymal stem cells; hUCSCs, human umbilical cord-stem cells; NSCs, neural stem cells; hUMSCs, human umbilical cord mesenchymal stem cells; ASCs, adipose mesenchymal stem cells; HAFSCs, human amniotic fluid stem cells; cAT-MSCs, canine adipose tissue-derived mesenchymal stem cells, MS, Multiple sclerosis.

MS models, particularly EAE, provide the principal experimental platform for evaluating the therapeutic effects of MSC-EVs on neuroinflammation and demyelination (117). Recent studies have shown that MSC-EVs can attenuate inflammatory responses in the CNS, regulate immune cells such as microglia and T cells, reduce demyelination, and protect neural cells from injury (118–120). The following sections summarize and elaborate on the role of MSC-EVs in CNS inflammatory diseases, such as encephalomyelitis, with an emphasis on their core therapeutic mechanisms rather than the general disease background (119, 120).

### 5.1 SC-EVs modulate immune responses

Immune-mediated attacks on neural tissues are the primary drivers of CNS damage in MS. Cytotoxic T-cell responses, including CD4^+^ and CD8^+^ T cells, can amplify neuroinflammation and exacerbate disease progression (117, 118). In contrast, regulatory subsets suppress inflammatory responses and protect the nervous system from damage. In addition to T cells, microglia and DCs play important roles in MS pathology. Studies have shown that MSC-EVs can protect the nervous system and alleviate MS by modulating the cells and their associated immune responses.

Foxp3^+^ Tregs are cells that play a key regulatory role in the immune system, characterized by their ability to suppress immune responses (121). Accumulating evidence suggests that MSC-EVs promote immune tolerance in EAE models by expanding Foxp3^+^ Tregs and reinforcing their suppressive phenotype (including upregulation of inhibitory receptors such as Lag-3), accompanied by a broader shift from pro-inflammatory to anti-inflammatory cytokine programs (106, 109, 112). However, this study did not explore these findings in depth and could not determine whether Foxp3^+^CD4^+^T cells mitigate inflammatory responses by expressing Lag-3.

Fathollahi et al. (106) reported that following MSC-EV treatment, an MS mouse model exhibited reduced inflammation scores and an increased frequency of Foxp3^+^CD25^+^ Treg cells, along with the upregulation of anti-inflammatory factors. Similarly, Mohammadzadeh et al. (109) found that treatment with hUMSC-EVs significantly increased the number of Foxp3^+^CD4^+^ T cells and Lag-3 expression and reduced inflammation scores in an MS mouse model. Vakili et al. (112) further showed that bone marrow mesenchymal stem cells (BMSC-EVs) dampened immune responses by reducing splenocyte proliferation, increasing the frequency of Treg cells, and reshaping the cytokine milieu (decreasing TNF-α, IL-1β, and IL-6 while increasing IL-10 and TGF-β), thereby reducing inflammation-induced CNS damage.

Dendritic cells (DCs), another vital component of the immune system, play a significant role in immune regulation and in antigen presentation. Manni et al. investigated the effects of human amniotic fluid SC-EVs (HAFSC-EVs) on the immune system and immune responses in a mouse model of MS. Their research revealed that HAFSC-EVs are taken up by conventional dendritic cell type 2 (cDC2), promoting the transformation of DCs into a tolerogenic phenotype. Tolerogenic DCs exhibit reduced pro-inflammatory cytokines and pro-inflammatory activity. Moreover, these tolerogenic DCs can enhance Foxp3+ Treg expression, thereby suppressing immune responses and protecting the nervous system. Reis et al. discovered that EV-encapsulated miR-21-5p acts as an effective immunomodulator (99). When delivered to dendritic cells (DCs), this enriched miRNA targets the chemokine receptor CCR7, thereby physically blocking DC migration toward the lymph node chemokine CCL21 (99). This blockade effectively prevents the activation of self-reactive T cells while simultaneously suppressing DC maturation and pro-inflammatory cytokine release, thereby promoting their transition to a tolerogenic state (99).

Furthermore, EVs preconditioned with deferoxamine (EVDFO) have demonstrated significant potential for immune regulation (55). Park et al. (55) reported that in an MS mouse model treated with EVDFO, Foxp3+ Treg expression was increased. This augmented Treg population helps curb excessive immune responses, thereby effectively modulating the inflammatory system, alleviating neuroinflammation, and protecting the CNS from autoimmune attacks (55). This finding further supports the potential of EVDFO to regulate immune balance and mitigate the symptoms of autoimmune diseases, providing robust scientific evidence for the development of novel immunoregulatory therapies (55).

### 5.2 SC-EVs suppress inflammatory responses and alleviate damage

Inflammation triggered by various factors is the primary cause of neural tissue damage and functional impairment in patients with MS. By modulating the immune system, MSC-EVs primarily exert anti-inflammatory effects by targeting microglial and cytokine pathways in MS models. Ahmadvand Koohsari et al. (107) investigated the effects of human umbilical cord mesenchymal stem cell-derived exosomes (hUCMSC-EVs) in a mouse model of EAE. Their results demonstrated that hUCMSC-EVs can reduce pro-inflammatory cytokines in the nervous system, such as IL-17a, TNF-α, and IFN-γ, while increasing anti-inflammatory cytokines, such as IL-4 and IL-10, to mitigate inflammatory responses (107). In addition, hUCMSC-EVs decrease the infiltration of inflammatory cells, including leukocytes, and modulate T cell activity (107). Ahmadvand Koohsari et al. (107) further observed that hUCMSC-EVs improved inflammatory indices and reduced inflammation scores in animal models. In addition to hUCMSC-EVs, hADSC-EVs also suppressed inflammatory responses. Jafarinia et al. (105) revealed that treating MS mouse models with hADSC-EVs significantly improved inflammation scores and reduced inflammatory cell infiltration. Similarly, Laso-García et al. (115) showed that hADSC-EVs could reduce inflammatory cell infiltration, modulate neuronal activity, and promote microglial transformation into an anti-inflammatory phenotype in EAE. In mice treated with hADSC-EVs, the plasma levels of Th1 and Th17 cytokines were significantly reduced, indicating a marked improvement in inflammation (115). Preconditioned SC-EVs also exerted anti-inflammatory effects. Park et al. (55) found that EVs from canine adipose tissue-derived mesenchymal stem cells (cAT-MSCs) pretreated with deferoxamine (DFO) promoted microglial transformation into the anti-inflammatory M2 phenotype and regulated Treg responses in EAE mice, thereby reducing inflammatory responses and protecting the nervous system. Microglia are the primary immune cells in the CNS that play a central role in neuroinflammatory responses. Traditionally, activated microglia during neuroinflammation have been characterized as differentiating into pro-inflammatory M1 and anti-inflammatory M2 microglia. Li et al. (50) demonstrated that BMSC-EVs could significantly alleviate inflammatory responses in an EAE rat model. BMSC-EVs reduce inflammatory cell infiltration, promote the expansion of M2 microglia and their associated anti-inflammatory cytokines (such as IL-10 and TGF-β), and simultaneously inhibit the expression of M1 microglia and related pro-inflammatory cytokines (50). As research on EVs deepens and technologies mature, artificially engineered EVs can exert anti-inflammatory effects in the treatment of CNS inflammation. Wang et al. developed a type of TAxI-EVs, namely UMSC-TAxI-EVs. Studies have shown that UMSC-TAxI-EVs can more rapidly and efficiently modulate inflammatory responses, reduce the number of activated glial cells, and promote glial cell transformation into an anti-inflammatory phenotype owing to their precise targeting (54). Moreover, UMSC-TAxI-EVs can upregulate the expression of anti-inflammatory factors, such as IL-4, IL-10, TGF-β, and IDO-1, while do pro-inflammatory factors (54). MicroRNAs (miRNAs) are non-coding, single-stranded RNA molecules that reside within SC-EVs and exert various biological effects. Shi et al. discovered that miR-181a-5p within MSC-EVs specifically targets and binds to USP15, thereby modulating the RelA/NEK7 axis to inhibit microglial inflammation and pyroptosis. In summary, SC-EVs exert anti-inflammatory effects by modulating inflammatory cells and factors, promoting the secretion of anti-inflammatory factors, and regulating related inflammatory mechanisms through their cargo content.

### 5.3 SC-EVs exert neuroprotective effects

Encephalomyelitis can trigger inflammatory responses in the CNS, leading to various forms of nervous system damage, including neuronal death and injuries. One of the most pronounced forms of damage is the demyelination of the optic nerve. Myelin, produced by oligodendrocytes, ensheathes nerve fibers and functions as an insulator to accelerate signal conduction. Neuroinflammation can lead to myelin destruction, thereby impeding signal transmission and resulting in functional impairment throughout the body. Recent studies have shown that SC-EVs can protect and repair neural function by safeguarding myelin, reducing demyelination, and enhancing the function of oligodendrocytes.

In EAE, SC-EVs appear to converge on a remyelination module by preserving oligodendrocyte lineage cells. Regardless of EV origin (adipose, placenta, or deferoxamine-preconditioned MSCs), administration consistently reduces demyelinating pathology and stabilizes myelin integrity in the CNS (25, 55, 105). This convergence is explained by complementary mechanisms, including dampened inflammatory influx (reduced leukocyte infiltration) that alleviates ongoing myelin injury (105), coupled with direct trophic support of oligodendrocyte survival and maturation and reduced apoptosis (25, 105). Thus, myelin regeneration can be considered a common therapeutic outcome of SC-EV interventions. By maintaining the integrity of the oligodendrocyte lineage, SC-EVs promote myelin repair in demyelinating neuroinflammation, such as in EAE (25).

Building on these endogenous reparative mechanisms, next-generation engineered EVs offer a strategy to further amplify the remyelinating potential of the CNS (108). Xiao et al. (108) found that EVs secreted by neural stem cells (NSCs) engineered to highly express the ligand PDGF-A could achieve precise drug delivery. These EVPs enhance oligodendrocyte targeting. Loading substances that promote oligodendrocyte development, such as triiodothyronine (T3), onto EVPs can increase oligodendrocyte survival and promote myelin regeneration and repair (108).

Collectively, SC-EVs exert immunomodulatory and neuroprotective effects in EAE models. They attenuate pro-inflammatory cytokine signaling, promote Treg cell expansion, bias microglia toward an M2-like state, and support oligodendrocyte survival and remyelination.

## 6 The therapeutic modulatory effects of SC-EVs in SLE

SLE is characterized by widespread loss of tolerance and multi-organ injury. Given the limitations of current immunosuppressive therapies, MSC-EVs have emerged as a promising cell-free strategy for restoring immune homeostasis in patients with SLE. Preclinical data, predominantly from the lupus-prone mouse model (MRL/lpr), have demonstrated that MSC-EVs potently ameliorate systemic inflammation and reduce autoantibody titers. Although these models rarely exhibit frank CNS demyelination, as seen in human NPSLE, the resolution of systemic autoimmunity may indirectly benefit the CNS by mitigating peripheral cytokine surges that compromise the BBB. The reference lists of eligible studies were manually screened to identify additional research. This process yielded 15 studies (24, 56, 57, 97, 103, 104, 119, 120, 122–128) published between 2016 and 2025 that explored the effects of MSC-EVs in lupus models. The majority (24, 56, 57, 97, 103, 120, 122–124, 126) used lupus-prone murine models, such as MRL/lpr and NZB/W F1 mice (*n* = 7) (57, 97, 103, 120, 123, 124, 126), and pristane-induced models (111, 113, 117). *In vitro*/*ex vivo* systems include peripheral blood mononuclear cells (PBMCs)-derived CD4^+^ T cells (119, 127), B cells from SLE patients (112, 121), THP-1(human monocytic cell line) macrophages (114, 118), and splenic mononuclear cells from MRL/lpr mice (123).

The experimental design relied on *in vivo* mouse models for therapeutic evaluation, complemented by *in vitro* validation using immune cells isolated from these mice. Biomarker assessments have focused on lupus-associated pro-inflammatory cytokines (TNF-α, IL-6, IL-17A, IFN-γ), anti-inflammatory cytokines (IL-10, TGF-β), T cell subsets (Th17/Treg balance, Foxp3^+^ CD4^+^ T cells), macrophage polarization (M1/M2), B cell activation, autoantibody levels (anti-dsDNA), and renal immune complex deposition (97, 103, 104, 126, 128). Collectively, these studies demonstrate that MSC-EVs possess potent immunomodulatory properties that alleviate systemic inflammation and restore immune homeostasis. Functionally, MSC-EVs suppress pathogenic T-cell activation, inhibit Th17 differentiation, promote Treg expansion, and modulate inflammatory signaling, notably the IL-6/STAT3/IL-17 axis (97, 119, 126, 127).

MSC-EVs also regulate macrophage polarization by inhibiting the M1 phenotype and promoting M2 differentiation, thereby facilitating inflammatory resolution (56, 57, 122, 123, 125). In the humoral immune compartment, MSC-EVs attenuate B cell hyperactivation, reduce plasma cell differentiation, and decrease circulating autoantibody levels, ultimately limiting immune complex formation and deposition (103, 104, 126, 128). These effects translate to end-organ protection, particularly in lupus nephritis, by reducing glomerular deposition, suppressing renal inflammation, enhancing podocyte survival, and improving function and survival (97, 103, 120, 126, 128).

Mechanistically, MSC-EVs deliver cargo, including microRNAs (miR-19b, miR-16-5p, miR-146a-5p, miR-16/miR-21, and miR-20a), regulators of the HIF-1α pathway, apoptotic components, and enzymes, thereby modulating immune activation and injury pathways (24, 56, 97, 119, 120, 124, 127). Despite the heterogeneity in EV origin (bone marrow-, umbilical cord-, and adipose-derived), dosing, and routes of administration, these findings support MSC-EVs as a cell-free strategy for immune regulation and organ protection in SLE. The detailed experimental design and outcomes are summarized in Table 2. SLE is a chronic autoimmune disease characterized by dysregulated innate and adaptive responses, excessive autoantibody production, immune complex deposition, and multi-organ damage. Patients present with diverse manifestations involving the skin, joints, kidneys, and CNS. Owing to phenotypic heterogeneity and complex mechanisms, current treatments (immunosuppressants and biologics) are limited by incomplete efficacy and adverse effects, respectively. Emerging evidence suggests that MSC-EVs suppress pathological immune activation, rebalance cell subsets, and protect organs. The following sections summarize their immunomodulatory roles based on recent evidence available.

**Table 2:** Preclinical and clinical stroke studies assessing the effect of EVs from MSCs on the regulation of systemic lupus erythematosus.

References	MSC-EVs source	Recipient cells	Cargos	Model	Main functions
Tu et al. (119)	hUCMSCs	CD4+ T cells	miR-19b	*In vitro* model of CD4+ cells of PBMC cells	Promote the expression of T cells by expressing miR-19b to inhibit the level of KLF13 and improve Th17/Treg cells regulation
Wei et al. (120)	ADSCs	Glomerular mesangial cell	miR-20a	B6.MRL/lpr mice	Increased podocin and nephrin, and higher autophagosomes. Reduced podocyte damage through autophagy
Zhang et al. (24)	BMSCs	Macrophages	miR-16, miR-21, PDCD4, and PTEN	C57BL/6 mice were induced with pristane oil	Reduced pro-inflammatory cytokines and increased anti-inflammatory cytokines
Zhao et al. (104)	hUMSCs	B cell	miR-155 and SHIP-1	PBMCs of SLE patients	Reduced B-cell hyperactivation. Reduced cytokine levels of IL-16, IL-10, INF-γ, IL-17, 1L-4, and TNF-α. High levels of IL-6- due to paracrine effect from MSCs
Chen et al. (122)	hUMSCs	Macrophages	Not available	C57BL/6J mice (SPF) induced by pristine to develop DAH	Enhances the polarization of M2 macrophage to alleviate DAH. Improved phagocytosis of macrophages in DAH. Facilitated the transformation of macrophages from M1 to M2 phenotype.
Sun et al. (57)	hUMSCs	Macrophages and T cells	Not available	pTHP-1 macrophages C57BL/6lpr-/-(B6.lpr)	Macrophage proliferation, inflammation, and M1 polarization were inhibited post-treatment.
Xie et al. (123)	hUCMSCs	T cells and B cells	Not available	Splenic mononuclear cells of MRL/lpr mice	CD4+ T cells were inhibited in splenic mononuclear cells of MRL/lpr mice, thereby promoting Th17 cell differentiation and increasing IL-17 and TGF cytokine levels.
Wang et al. (124)	BMSCs	CD4+T cells	apoVs	MRL/Ipr mice. Apoptosis induced with staurosporine (STS)	Apoptotic vesicles (ApoVs) derived from apoptotic BMMSCs. Decreased T cells in lymphoid tissue. Increased CD4^+^ T effector cells. Decreased CD4^+^ T naïve cells. Reduction in IFNγ^+^ and CD4^+^ T cells. Increased Foxp3^+^ CD4^+^ T cells effector. Reduction in lymphoproliferation. Reduced levels of anti-dsDNA and IgG
Chen et al. (56)	hUCMSCs	Macrophage	miR-146a-5p	C57BL/6J mice were induced with 0.5 mL pristane.	Exosomes promote the polarization of M2 macrophages via miR-146a-5p/NOTCH1/HIF-1α axis, inhibit the expression of NOTCH1, IL-1β, and iNOS, thereby reducing inflammation and bleeding.
Dou et al. (125)	BMSCs	Macrophage	tsRNA-21109	TPH-1 cell line + PMA to induce macrophage differentiation	Decrease in M1/M2 polarization. Reduction in TNF-α and IL-1β. Changed the TRF expression of M1 macrophages.
Sonodaet al. (126)	BMSCs	Tissue	Not available	Immunocompromised NOD-SCID mice BM-MSCs derived from NOD-SCID mice.	Reduced the peripheral autoantibody levels, renal functions, and levels of CD4^+^ IL-17^+^ IFN-gamma, and increased CD4^+^ CD25^+^ Foxp3+ in the PBMCs of the mice.
Zhang et al. (127)	ADSCs	T cells	miR-16-5p	*In vitro* model of CD4+ cells of PBMC cells	ADSC-EVs effectively deliver miR-16-5p, which targets and downregulates LATS1 expression. This regulation restores the Th17/Treg balance and modulates cytokine expression.
Liao et al. (128)	MSCs	SLE-B cells	anti-inflammatory protein IDO1	MSCs induced by five μM rapamycin.	Rapa-SLE-EV effectively suppresses plasma inflammatory cytokines, protects renal function, alleviates pathological damage, and reduces glomerular immune complex deposition
Li et al. (97)	hUCMSCs	T cell and B cell	IL-6/STAT3/IL-17 signaling pathway	16-week-old MRL/lpr mice were treated with hUCMSC-EVs.	hUCMSC-EVs modulate the Th1/Th17/Treg imbalance and suppress DNT cells and plasma cells. with HIF-1α suppression by miRNAs further contributing to reduced renal inflammation and fibrosis (152).
Choi et al. (103)	ADSCs	Macrophages	TGF-β1 miR-155-5p miR-142-3p	NZB/W F1 mice were treated with EVs derived from 1.33 × 10^6^ iMSCs and with EVs derived from 1.33 × 10^6^ iMSCs primed with conditioned medium, respectively.	Compared with ASC-EVs, CM-EVs more potently elevated TGF-β1 secretion and the levels of miR-155-5p and miR-142-3p, extended survival, reduced anti-dsDNA antibodies, and ameliorated renal injury.

EVs, extracellular vesicles; MSCs, mesenchymal stem cells; ADSCs, adipose tissue-derived MSCs; BMSCs, bone marrow mesenchymal stem cells; UCMSCs, umbilical cord mesenchymal stem cells; miR, microRNA; KLF13, Kruppel-like factor 13; Treg, regulatory T cells; Th17, T helper 17 cells; IL, interleukin; TNF, tumor necrosis factor; TGF, transforming growth factor; SLE, systemic lupus erythematosus; B6.MRL/lpr, lupus-prone mouse model; DAH, diffuse alveolar hemorrhage.

### 6.1 Restoration of Treg/Th17 and Th1/Th2 homeostasis

SLE disrupts T-cell homeostasis, with excessive Th17 activation and impaired Treg function linked to disease activity and damage in SLE. Th1/Th17 cytokines amplify inflammation downstream of type I interferon signaling, whereas Th17/Treg imbalance indicates disease severity. Preclinical studies have shown that MSC-EVs modulate CD4^+^ T-cell fate via microRNAs and cargo, suppressing Th17 responses and promoting Treg programs. MSC-EVs directly regulate T cells through miRNA delivery. Multiple miRNAs (miR-146a, miR-10a, miR-21, miR-19b) regulate the Th17/Treg axis (119, 129, 130).

MSC-EV-associated miRNAs act within T cells to reshape effector vs. regulatory differentiation programs. Tu et al. (119) reported that miR-19b-rich umbilical cord-derived mesenchymal stem cell exosomes can be efficiently internalized by CD4^+^ T cells and promote the restoration of the Th17/Treg axis balance by targeting Kruppel-like factor 13 (KLF13) expression. miR-16 (together with miR-21) primarily affects Th17/Treg axis homeostasis by regulating macrophage polarization toward an anti-inflammatory phenotype (109, 120, 126). Zhang et al. (127) reported that adipose-derived MSC exosomes deliver miR-16-5p to CD4^+^ T cells, where it targets and downregulates large tumor suppressor kinase 1 (LATS1).

In addition to T cell-intrinsic effects, MSC-EVs suppress inflammatory signaling. In the MRL/lpr mouse model, injection of hUCMSC-EVs suppressed sustained activation of the IL-6/STAT3/IL-17 signaling pathway and pro-inflammatory cytokine levels, and diminished Th17 cell differentiation and infiltration in renal tissue. Concurrently, UC-MSC-EVs significantly corrected Th1/Th17/Treg immune dysregulation and suppressed the abnormal expansion of double-negative T cells and plasma cells (97). Sonoda et al. (126) further demonstrated that BMSC-EVs decreased peripheral IL-17^+^/IFN-γ^+^ T cells while increasing Foxp3^+^ Tregs, improving renal function, and reducing autoantibodies. However, MSC-EV-mediated effects on T cells are not uniformly suppressive. Xie et al. (123) found that hUCMSC-EVs promoted Th17 differentiation in MRL/lpr splenocytes, enhancing IL-17/TGF-β.

Apoptotic vesicles (ApoVs) from MSCs, which contain phosphatidylserine, regulate T cell activation. Wang et al. (124) demonstrated that ApoVs generated from apoptotic BMSCs reduced lymphoproliferation, inhibited the production of Th17 cells and related cytokines, and expanded Foxp3^+^ CD4^+^ T-cell populations in MRL/lpr mice. In addition, *in vitro* and across various inflammatory models, ApoVs can reduce the frequency of IFN-γ^+^CD4^+^ T cells (Th1-related) by directly interfering with CD4^+^ T cell activation and, to some extent, limiting the differentiation of Th1 and Th2 cells into effector cells (124, 131). Overall, MSC-EVs enhanced Treg function while suppressing Th17 function via direct miRNA reprogramming and indirect cytokine modulation.

### 6.2 Restoration of B-cell homeostasis

MSC-EVs regulate B cell activation, differentiation, and antibody production in SLE by delivering cargo, including miRNAs and proteins. Zhao et al. (104) reported that hUCMSC-EVs deliver miR-155 to B cells. This miRNA inhibits B cell hyperactivation by targeting SHIP-1 and reduces the secretion levels of multiple cytokines (IL-6, IL-10, IFN-γ, IL-17, IL-4, TNF-α). Liao et al. (128) showed that rapamycin-preconditioned MSC-EVs enriched with indoleamine 2,3-dioxygenase 1 (IDO1) targeted SLE B cells suppressed cytokine production, reduced glomerular complexes, and improved renal injury. MSC-EVs influence B cells through various pathways. Li et al. (97) reported that hUCMSC-EVs modulate the IL-6/STAT3/IL-17 axis, inhibiting Th17 and plasma cell expansion, and decreasing autoantibody levels.

Evidence from *in vivo* models suggests that the effects of MSC-EVs on B cells are closely linked to their capacity to reshape the surrounding immune microenvironment. Sonoda et al. (126) observed that BMSC-EVs treatment reduced peripheral autoantibody levels in NOD-SCID mice, paralleled by a decrease in IL-17^+^ and IFN-γ^+^ CD4^+^ T cells and an increase in Foxp3^+^ regulatory T cells. Xie et al. (123) noted that hUCMSC-EVs alter splenic immune composition, modulating CD4^+^ T-B interactions and Th17-associated cytokines such as IL-17 and TGF-β.

### 6.3 SC-EV promotes macrophage polarization from M1 to M2 and enhances efferocytosis

Endocytosis refers to the process by which macrophages and other phagocytes programmatically and silently clear apoptotic cells, serving as a key mechanism for maintaining tissue homeostasis and promoting inflammation resolution (132, 133). This process primarily relies on phagocytes recognizing “eat-me” signals (such as phosphatidylserine) on the surface of apoptotic cells, followed by uptake and degradation mediated by receptors such as MerTK and TIM-4, along with bridging molecules such as C1q and MFG-E8 (134). In addition to clearing cellular debris, phagocytosis suppresses NF-κB signaling pathways, further enhancing the production of anti-inflammatory mediators such as IL-10 and TGF-β (134, 135).

In SLE, inadequate clearance of apoptotic cells coexists with persistent inflammation, hindering the restoration of immune homeostasis (133, 136). Increased lymphocyte apoptosis coincides with impaired phagocytic function (e.g., C1q deficiency or MerTK cleavage and shedding), resulting in clearance demands far exceeding phagocytic capacity (135). Unremoved apoptotic cells undergo secondary necrosis, releasing nucleic acid autoantigens that persistently activate the TLR7/TLR9 pathways in macrophages and other innate immune cells (137). This induces excessive type I interferon production and promotes NETosis (133, 136).

In this pathological context, SC-EVs may promote the resolution of inflammation and facilitate the recovery of phagocytic processes by modulating macrophage polarization and its associated signaling pathways (138). Zhang et al. (24) demonstrated that BMSC-EVs deliver miR-21 and miR-16 to recipient macrophages. These miRNAs suppress PDCD4 and PTEN expression, thereby relieving the inhibitory constraints on M2 polarization, reducing pro-inflammatory cytokine production, and preferentially shifting macrophages toward a reparative phenotype (24). Subsequent research by Chen et al. (56) further demonstrated that miR-146a-5p-rich hUCMSC-EVs inhibit the NOTCH1 signaling pathway, thereby accelerating macrophage M2 phenotype conversion and reducing IL-1β/iNOS levels. In a pristinol-induced diffuse alveolar hemorrhage (DAH) model, hUCMSC-EVs promoted M1-to-M2 conversion and enhanced phagocytic clearance of tissue debris, accelerating the recovery of inflamed tissues (122). Multiple independent studies have further supported the role of SC-EVs in reprogramming macrophage function. Sun et al. (57) reported that hUCMSC-EVs inhibit the proliferation, activation, and M1 polarization of M1 macrophages in pTHP-1 cells and B6.lpr mice. Dou et al. (125) discovered that tsRNA-21109 in BMSC-EVs participates in pro-inflammatory cytokines and reshapes M1-associated tRF regulatory networks. Additionally, signaling alterations mediated by TGF-β1, miR-155-5p, and miR-142-3p contribute to the anti-inflammatory reprogramming of phagocytosis in an NZB/W F1 mouse model (103). Collectively, these findings suggest that SC-EVs may restore defective efferocytosis in SLE by promoting macrophage M2 polarization and enhancing the clearance of apoptotic cells.

### 6.4 Improving kidney function

In lupus nephritis (LN), MSC-EVs regulate immune responses, clear complexes, and maintain glomerular homeostasis in the kidneys of patients. Umbilical cord MSC-EVs transfer miR-20a to mesangial cells and podocytes, thereby promoting autophagy and supporting structural proteins (120). In MRL/lpr mice, MSC-EVs suppress renal inflammation by correcting Th1/Th17/Treg dysregulation via the IL-6/STAT3/IL-17 axis (97). BMSC-EVs reduce autoantibody levels and improve renal function via immunomodulation (126). Immune complex–mediated glomerular injury, a central pathogenic feature of LN, is effectively attenuated by EVs. Rapamycin-induced MSC-EVs enriched in IDO1 reduce inflammatory cytokine production, decrease glomerular immune complex deposition, and alleviate renal pathological injury (128).

Similarly, apoptotic vesicles derived from BMSCs reduced anti-dsDNA antibody and IgG levels, thereby suppressing immune complex–driven renal inflammation (124). Consistent with these effects, EV treatment markedly reduced the glomerular deposition of IgG, C3, IgM, and C1q and improved renal functional parameters, including serum creatinine levels and proteinuria (24, 120). In addition to intrarenal immune mechanisms, the gut–kidney axis has been implicated in the pathogenesis of LN. Valiente et al. (139) demonstrated in the NZM2410 lupus nephritis model that gut microbial dysbiosis is correlated with increased renal immune complex deposition and CD206^+^ macrophage infiltration. EV-mediated modulation of gut microbial homeostasis may provide a mechanistic basis for immunotherapy targeting the gut–kidney axis in patients with lupus nephritis. Young et al. (140) demonstrated that EV-associated miRNAs modulate target organ inflammation in a humanized mouse model of SLE, thereby decelerating disease progression. HIF-1α participates in renal hypoxic stress and fibrosis in patients with SLE (141). Urinary-derived EV-miRNAs (such as miR-135b-5p, miR-107, and miR-31) alleviate kidney damage in LN by inhibiting HIF-1α (142). Targeting HIF-1α, which is regulated by EVs, reduces hypoxia-induced signaling pathways and promotes renal recovery, providing a mechanistic basis for the renal protective effects of EVs in LN pathology (141, 142).

Overall, MSC-EVs similarly rebalance dysregulated immunity by reshaping T cell, B cell, and myeloid compartments in SLE. These effects are commonly attributed to vesicular bioactive cargo, including specific miRNAs and proteins, which constrain pathogenic inflammation while reinforcing regulatory programs and tissue-protective pathways.

## 7 Overlapping molecular mechanisms and modes of action of SC-EVs in SLE and EAE

**Figure 2:**
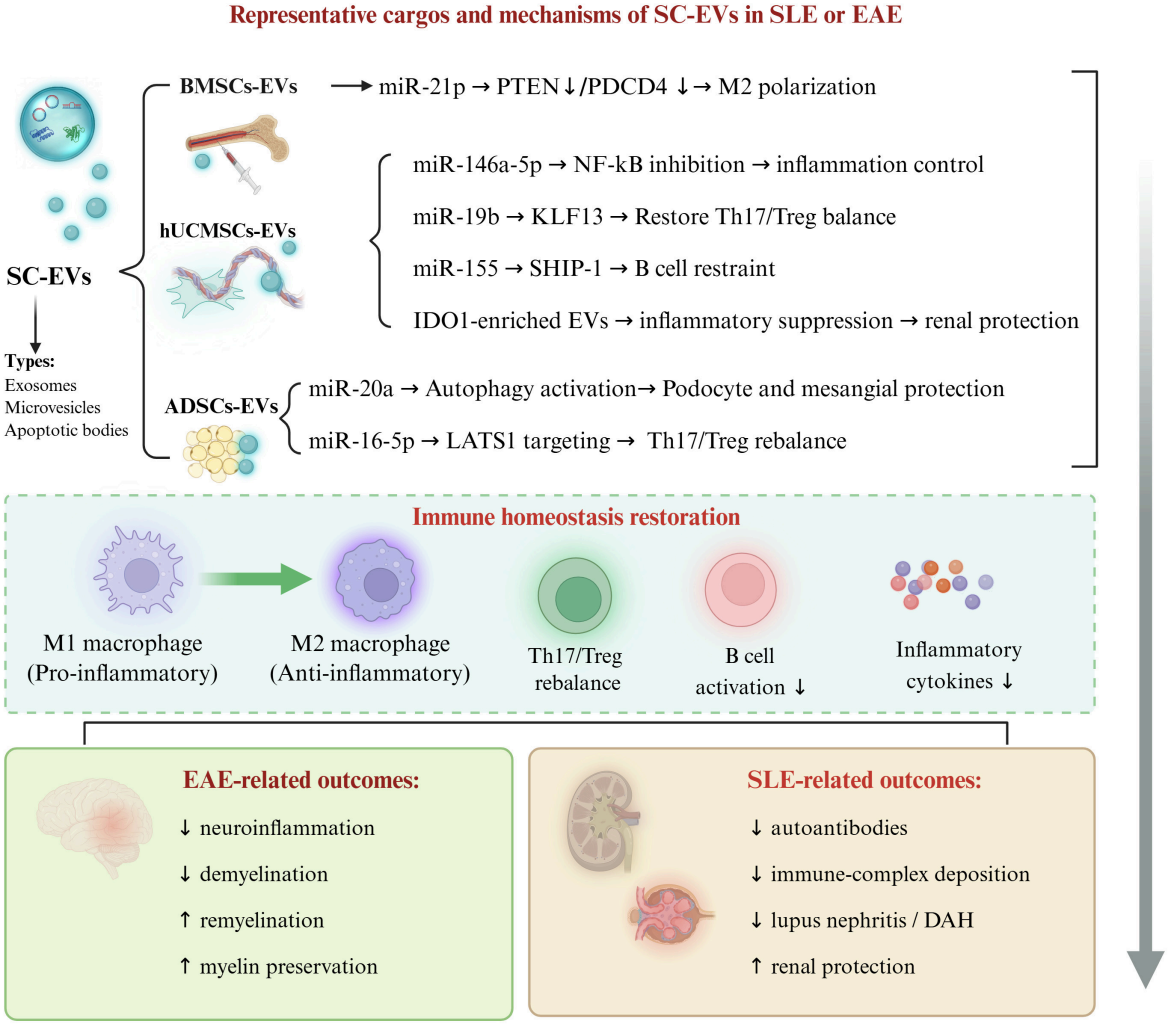
Representative cargos, immunomodulatory mechanisms, and disease-related outcomes of SC-EVs in SLE or EAE. Bioactive cargos and immunomodulatory outcomes of SC-EVs in SLE and MS/EAE. SC-EVs from diverse cellular sources, including BMSCs, UMSCs, and ADSCs, deliver functional cargos such as miRNAs, tsRNAs, and proteins including IDO1 to recipient immune cells. These mediators regulate key signaling nodes to restore immune homeostasis by driving M1-to-M2 macrophage repolarization, rebalancing Th17/Treg ratios, and restraining B-cell hyperactivation. Shared therapeutic effects include systemic inflammation relief, while disease-specific outcomes include enhanced remyelination and myelin preservation in MS/EAE, and reduced immune-complex deposition and renal protection in SLE. ADSC, adipose-derived stem cell; BMSC, bone marrow mesenchymal stem cell; EAE, experimental autoimmune encephalomyelitis; EVs extracellular vesicles; hUCMSCs, human umbilical cord mesenchymal stem cells; IDO1, indoleamine 2,3-dioxygenase 1; MS, multiple sclerosis; SC-EVs, stem cell-derived extracellular vesicles; SLE, systemic lupus erythematosus; Th17, T helper 17 cell; Treg, regulatory T cell; UMSCs, umbilical cord mesenchymal stem cells; KLF13, Krüppel-like factor 13; LATS1, large tumor suppressor kinase 1; miR, microRNA; NF-κB, nuclear factor kappa B; PDCD4, programmed cell death 4; PTEN, phosphatase and tensin homolog; SHIP-1, SH2 domain-containing inositol5′-phosphatase 1; MSC, umbilical cord mesenchymal stem cell.

**Figure 3:**
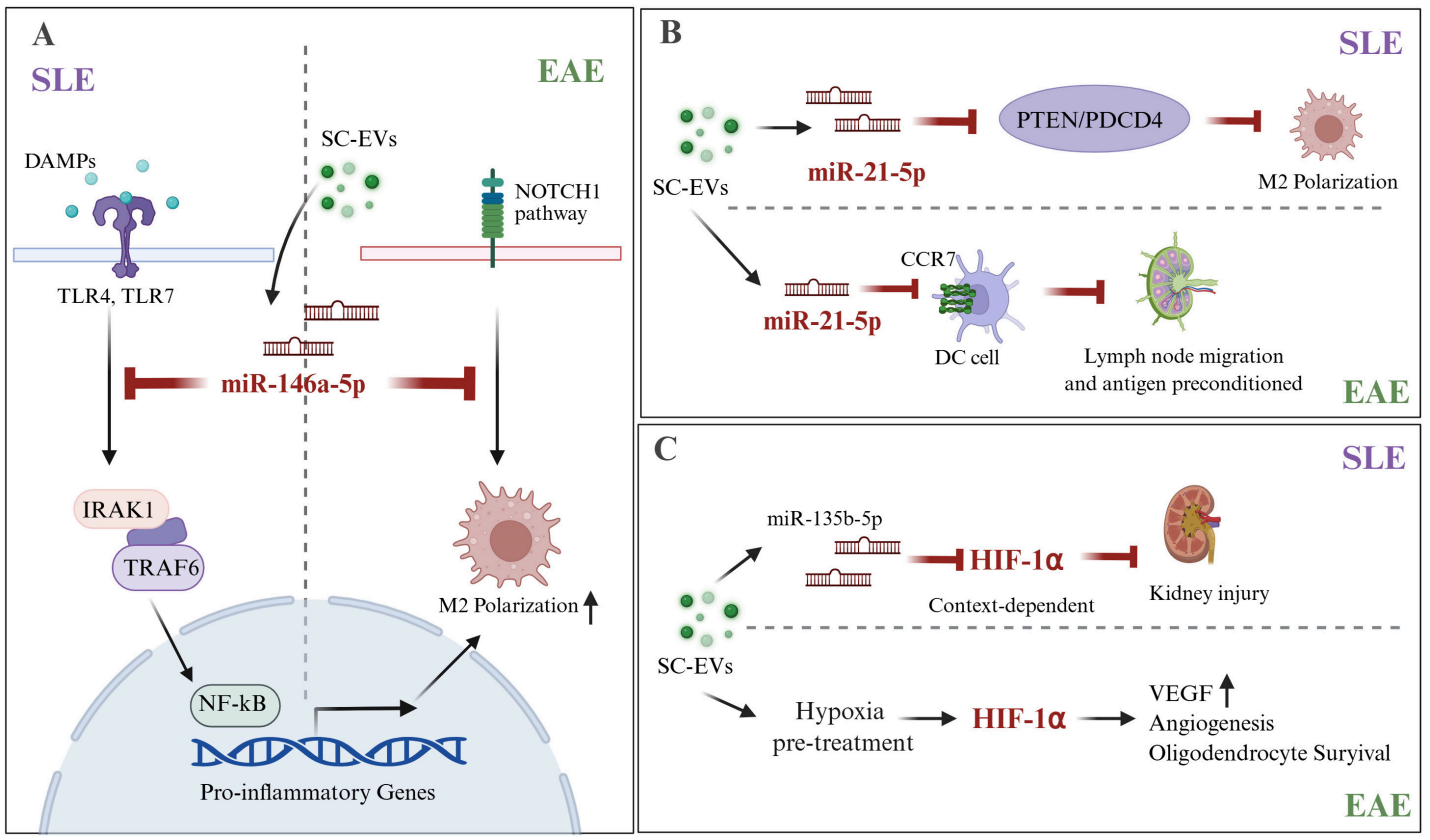
Shared molecular nodes underlying SC-EVs-mediated immunomodulation in SLE and EAE. Three primary pathways connect SC-EV signaling to the treatment of SLE and MS/EAE. **(A)** miR-146a-5p suppresses innate inflammatory amplification by targeting the IRAK1/TRAF6/NF-κB axis in SLE and promotes reparative M2 polarization via NOTCH1 signaling in MS models. **(B)** miR-21-5p exhibits context-specific effects: in SLE, it facilitates M2 polarization by releasing metabolic brakes via the PTEN/PDCD4 axis, whereas in MS, it restricts DC migration and antigen priming by targeting CCR7. **(C)** HIF-1α acts as a bidirectional regulator; its inhibition via miR-135b-5p alleviates renal injury in SLE, while its controlled activation (e.g., via hypoxia pretreatment) supports angiogenesis and oligodendrocyte survival during CNS repair in MS. CCR7, C-C chemokine receptor type 7; DAMPs, damage-associated molecular patterns; SLE, systemic lupus erythematosus; EAE, experimental autoimmune encephalomyelitis; HIF-1α, hypoxia-inducible factor-1 alpha; IRAK1, interleukin-1 receptor-associated kinase 1; NF-κB, nuclear factor kappa B; PDCD4, programmed cell death 4; PTEN, phosphatase and tensin homolog; SC-EVs, stem cell-derived extracellular vesicles; TRAF6, TNF receptor-associated factor 6; VEGF, vascular endothelial growth factor; TLR4/7, Toll-like receptors 4/7; TRAF6, TNF receptor-associated factor 6.

Despite affecting different primary organ systems, SLE and EAE share conserved molecular mechanisms that are mediated by EV cargos. From an immunopathological perspective, multi-organ involvement in SLE and CNS inflammation in MS converge on shared fundamental pathways, including dysregulated type I interferon responses, lymphocytic infiltration, and BBB disruption (65, 143–145). Crucially, this convergence was not diffuse but rather concentrated. Still, centers on a highly conserved core signature, anchored by microRNAs [notably microRNA-146a-5p (56, 113) and miR-21 (24, 99)] and metabolic regulators [HIF-1α (55, 142)] as shared pivotal immunomodulators. This substantial molecular overlap not only illuminates the shared fundamental pathophysiological processes of autoimmune diseases but also provides critical targets for developing broad-spectrum therapeutic strategies to mitigate tissue damage. Emerging studies have further demonstrated the therapeutic potential of engineered EVs for targeted cargo delivery, with encouraging results in EAE models. These findings establish exosomal miRNAs as fundamental mediators of cross-organ protective mechanisms and as viable therapeutic targets for the management of autoimmune diseases. For detailed information on mesenchymal SC-EVs in SLE and EAE research and overlapping targets and mechanisms in SLE and EAE, refer to Figures 1–3 and Tables 1, 2.

### 7.1 miR-146a-5p: a core negative regulator of innate inflammation in both diseases

miR-146a-5p is a highly conserved microRNA that plays a dual role in immune regulation and inflammatory control, and its dysregulation is closely associated with the pathogenesis of a wide range of autoimmune and inflammatory diseases (56, 146). As a canonical negative feedback regulator of innate immunity, the early upregulation of miR-146a-5p exerts a critical inhibitory effect on excessive inflammation by directly targeting the adaptor molecules TNF receptor-associated factor 6 (TRAF6) and interleukin-1 receptor-associated kinase 1 (IRAK1) within the Toll-like receptor (TLR)/NF-κB signaling pathway, thereby limiting the magnitude and duration of immune activation (56, 113). However, under chronic inflammatory conditions, persistent dysregulation of this miRNA disrupts immune tolerance, leading to sustained type I interferon activation, chronic immune imbalance, and progressive tissue damage. The expression of miR-146a-5p is precisely regulated at multiple levels, including transcriptional activation by NF-κB and other transcription factors, epigenetic modifications that affect its maturation, and intercellular communication via exosomal transport. Owing to its high stability in serum, miR-146a-5p has been extensively explored for its clinical potential as a diagnostic tool. It is also regarded as a therapeutic target that can be regulated via nanocarrier delivery systems (144, 145). Its pivotal role in immune regulation makes miR-146a-5p a promising candidate for precision medicine strategies in autoimmune diseases.

miR-146a-5p functions as a key regulatory molecule in SLE, modulating both innate and adaptive immune responses. In patients with SLE, miR-146a-5p expression is significantly upregulated in peripheral blood mononuclear cells, particularly in CD4+ T cells and monocytes, representing a compensatory mechanism to restrain excessive immune activation (147). Dysregulation of miR-146a-5p substantially contributes to the breakdown of immune tolerance, as its persistent overexpression leads to aberrant B cell activation and autoantibody production while simultaneously promoting T cell hyperactivity and Th1/Th17 polarization. Clinically, miR-146a-5p levels in the serum and peripheral blood correlate with disease activity, particularly renal involvement and neuropsychiatric manifestations (147). Current therapeutic strategies are exploring the potential of modulating miR-146a-5p expression using antisense oligonucleotides or mimetic approaches, with promising results in lupus-prone mouse models demonstrating reduced autoantibody titers and improved renal histology (146). Furthermore, EV-mediated delivery of miR-146a-5p represents an innovative approach for targeted immunomodulation in SLE, offering the potential for tissue-specific therapeutic interventions while minimizing systemic side effects. In a mouse model of SLE-associated diffuse alveolar hemorrhage, overexpression of miR-146a-5p decreased levels of M1 markers and inflammatory factors while increasing M2 marker levels in macrophages. This effect was reversed by NOTCH1 overexpression (56). hUCMSC-EVs reduced NOTCH1 expression, bleeding, inflammation, and M1 macrophage polarization, while elevating M2 macrophage polarization in the lungs of mice with diffuse alveolar hemorrhage. hUCMSC-EVs with miR-146a-5p inhibition increased NOTCH1 expression, worsened bleeding and inflammation, and augmented M1 macrophage polarization while decreasing M2 macrophage polarization in the lung tissues of mice with diffuse alveolar hemorrhage. These effects were abrogated by NOTCH1 silencing (56). These findings demonstrate that hUCMSC-EVs diminish NOTCH1 expression to accelerate M2 macrophage polarization through miR-146a-5p delivery, thereby alleviating SLE-associated diffuse alveolar hemorrhage in mice (56).

In EAE, miR-146a-5p plays a complex dual role as a key negative feedback regulator of innate immune responses (148). Its expression is significantly upregulated by inflammatory signals, such as NF-κB, which initially serves as a protective brake by targeting key signaling molecules, such as IRAK1 and TRAF6, to suppress excessive inflammation (149). However, under chronic disease conditions, this sustained upregulation becomes pathogenic, contributing to immune dysregulation by impairing regulatory T cell function and hindering CNS repair by inhibiting oligodendrocyte precursor cell differentiation, thereby obstructing the remyelination. Given these mechanisms, miR-146a-5p has emerged as a promising biomarker and therapeutic target. In this context, MSC-EVs have attracted attention as cell-free therapeutics due to their immunomodulatory and reparative properties, particularly through the delivery of microRNAs (miRNAs). Shahryari et al. (113) investigated the therapeutic potential of EVs enriched with miR-146a in EAE, a murine model of MS. Treatment with miR-146a-enriched EVs from human adipose-derived MSCs significantly attenuated reduced pro-inflammatory cytokines (TNF-α, IFN-γ, IL-17), and increased anti-inflammatory cytokines (IL-10, TGF-β) in splenocyte cultures and spinal cord tissues (100). These effects were accompanied by downregulation of IRAK1 and TRAF6 mRNA levels, indicating effective suppression of the NF-κB pathway (113). Histopathological analysis further confirmed diminished neuroinflammation and demyelination, along with enhanced myelin preservation in mice treated with miR-146a-enriched EVs (113). Collectively, these findings indicate that miR-146a-5p is a central regulator of neuroinflammation and a viable therapeutic target. The targeted delivery of miR-146a via MSC-EVs can simultaneously modulate immune responses and promote CNS repair, establishing an effective strategy to mitigate the progression of encephalomyelitis.

Existing evidence indicates that miR-146a-5p plays a pivotal regulatory role in the neuroinflammation-associated immune network in both SLE and EAE. Dysregulation of this pathway is associated with inflammatory amplification, immune imbalance, and tissue damage. The delivery of miR-146a-5p via MSC-EVs simultaneously modulated multiple immunoregulatory pathways, generating synergistic effects in both inflammation suppression and CNS repair. This approach demonstrates consistent therapeutic potential for disease progression in both SLE and MS. Specifically, in SLE, it alleviates alveolar hemorrhage by regulating NOTCH1-mediated macrophage polarization, whereas in encephalomyelitis, it reduces neuroinflammation and promotes myelin repair by suppressing the NF-κB pathway (56, 113). Moreover, the substantial overlap in downstream immunoregulatory effects between SLE and EAE suggests that a single miRNA cannot explain the therapeutic effects of MSC-EVs. The complex molecular signaling networks that they carry may collectively shape convergent immune reconstruction efficacy, and the underlying mechanisms require further investigation to elucidate these effects.

### 7.2 miR-21-5p: complementary mechanisms targeting adaptive immunity

Mechanistically, miR-146a-5p primarily attenuates innate inflammatory signaling by suppressing NF-κB activity, whereas miR-21-5p exerts synergistic regulatory effects on adaptive immune regulation through two complementary mechanisms (24, 99). This regulatory axis exhibits distinct cellular specificity, depending on the pathological context. Complementing innate regulation, miR-21-5p exerts synergistic effects on adaptive immunity through distinct cellular target. In SLE models, miR-21-5p primarily targets macrophages. It enhances macrophage survival by releasing metabolic suppression mediated by the PTEN/PDCD4 axis and drives their polarization toward a reparative M2 phenotype, thereby promoting the resolution of systemic inflammation (24). Conversely, in the context of EAE, the effector targets shift to dendritic cells (DCs). Here, miR-21-5p suppressed the expression of the chemokine receptor CCR7, effectively blocking the chemotactic pathway for DCs migration to the lymph nodes. This physical restriction prevents antigen presentation at the source, thereby avoiding the pathogenic activation of autoreactive T cells (99).

Although the therapeutic efficacy of miR-21-5p in EAE is primarily based on plausible *in vitro* mechanistic inferences, the direct anti-inflammatory action of miR-146a-5p *in vivo* provides a strong empirical foundation. These miRNAs collectively form complementary immune tolerance checkpoints. miR-146a-5p suppresses innate inflammatory storms, whereas miR-21-5p remodels the metabolic and spatial patterns of adaptive immunity. This synergy ultimately blocks T cell dysfunction, which is a core pathogenic process in SLE and MS. Collectively, these findings indicate that miR-21 orchestrates a dual protective program by coupling the metabolic reprogramming of innate immune cells with the spatial restriction of adaptive immune activation, reinforcing its central role in MSC-EVs-mediated immunomodulation.

### 7.3 Context-dependent regulation of the HIF-1α pathways

Beyond the miRNA network, HIF-1α pathways serve as common targets influenced by distinct regulatory mechanisms. MSC-EVs appear to regulate the HIF-1α pathway in a tissue-and microenvironment-specific manner. Hypoxia pre-treatment of mesenchymal stem cells induces HIF-1α expression, which yields EVs enriched in pro-angiogenic factors (150). These EVs exert anti-inflammatory effects by favoring M2 macrophage polarization, an approach that benefits SLE models (151). In contrast, under normoxic conditions, EVs may contain inhibitory miRNAs (e.g., miR-146a), rendering them better suited for suppressive functions in SLE (145). The cargo within MSC-EVs governs activation or suppression, depending on the disease context. EVs-associated miR-135b-5p can inhibit HIF-1α expression to block hypoxia-driven cellular changes and limit glomerular fibrosis (142). Conversely, in EAE lesions requiring neural regeneration, MSC-EVs can stimulate the HIF-1α signaling pathway, thereby supporting angiogenesis and oligodendrocyte survival (55, 93, 142). The combined effects of miRNAs and proteins promote metabolic reprogramming and aid in neuroprotection. In SLE, exosomes convey miRNAs, such as miR-135b-5p, miR-107, and miR-31, which bind to the3′ untranslated region 3′UTR) of HIF-1α to repress its transcription (142), thereby alleviating inflammation and fibrosis. EVs can also activate autophagy or deliver IDO1 to suppress downstream inflammatory pathways (128) and preserve renal function. Indirect pathways, such as the limitation of VEGF-mediated vascular permeability, substantially contribute to this process. The lipid bilayer of EVs allows them to detect microenvironmental signals (e.g., pH, reactive oxygen species levels, or inflammatory cues) and selectively release cargo. However, unoptimized MSC-EVs may pose certain risks. In SLE, excessive HIF-1α activation could worsen fibrosis, whereas in MS, HIF-1α inhibition may impair repair processes. Thus, genetically engineering MSC-EVs can optimize their therapeutic efficacy (e.g., hypoxia pre-treatment for MS or inflammatory signal pre-treatment for SLE) (78). Concurrently, ongoing and future clinical trials of MSC- or EVs-based therapies must monitor HIF-1α levels to enable personalized EVs treatment regimens. Despite these contrasting upstream regulatory strategies, the downstream therapeutic outcomes are conserved. In both the BBB in MS and the glomerular filtration barrier in SLE, MSC-EVs modulate VEGF signaling to reduce endothelial permeability and prevent leakage of inflammatory mediators (85). This functional duality precludes a uniform strategy for activating or inhibiting HIF-1α. The pathway exerts opposing effects depending on the disease context and stage (78, 93). Therefore, effective EV-mediated regulation of HIF-1α requires precise control over the timing and cellular targets. In EAE, HIF-1α activity closely tracks the disease phases. During acute relapses, elevated HIF-1α levels are associated with pro-inflammatory polarization of microglia and macrophages, excessive VEGF production, and blood–brain barrier disruption. EVs engineered to inhibit HIF-1α, such as those carrying miR-135b-5p, miR-146a-5p, or miR-31, align well with this inflammatory setting by reducing vascular leakage and restraining immune cell infiltration (56, 113). Once inflammation resolves, moderate HIF-1α activity supports angiogenesis, oligodendrocyte precursor cell survival, and remyelination (93). Hypoxia-preconditioned EVs recapitulate the reparative signaling profile and facilitate tissue recovery (55). In SLE, particularly in LN and neuropsychiatric involvement, sustained HIF-1α activation coincides with chronic hypoxia, metabolic reprogramming, persistent cytokine production and progressive fibrosis (78, 142). These features predominate in the later stages of the disease. Therefore, EVs strategies that maintain HIF-1α suppression are more consistently aligned with therapeutic objectives in this context. Targeted and microenvironment-responsive EVs designs require further refinement. Surface modifications can direct preferential uptake by the inflamed endothelium, oligodendrocyte precursor cells, or specific immune subsets according to the disease phase. Triggered cargo release in response to oxidative stress or metabolic cues enables dynamic pathway modulation without imposing fixed directional bias.

### 7.4 Limitations and challenges

Despite these advances, this study has several limitations. Methodologically, the SC-EVs are heterogeneous. The significant variability in their source cells, coupled with the lack of standardized definitions, dosages, and administration protocols, makes it difficult to replicate the results and limits their potential for large-scale production. Biologically, key mediators such as HIF-1α can exert context-dependent effects across different tissues, disease stages, and pathological environments, sometimes producing opposing outcomes. This variability limits the universal applicability of the proposed mechanism. In addition, shared multifunctional targets may increase the risk of off-target effects or unintended immune modulation in the absence of precise regulatory controls. Pharmacokinetic challenges, including poor tissue specificity and inefficient systemic delivery, necessitate advanced surface engineering techniques for effective CNS and peripheral targeting of nanoparticles. The current evidence base remains predominantly preclinical, with models failing to fully replicate the chronicity, heterogeneity, and therapeutic windows of human diseases. However, the long-term efficacy of repeated EVs administration in chronic autoimmune diseases remains unclear. Future breakthroughs may depend on standardized production and precise EVs engineering to facilitate clinical translation.

## 8 Conclusion

This review provides a comprehensive overview of the emerging paradigm of exosome-mediated molecular regulation in autoimmune diseases, emphasizing their cross-organ protective roles. MSC-EVs have emerged as sophisticated biological carriers that deliver therapeutic cargo to target tissues. They demonstrated significant efficacy in both SLE and MS. The mechanistic basis of these protective effects involves dual pathways. EV-associated molecules modulate vascular endothelial growth factor signaling to promote tissue revascularization and stabilize hypoxia-inducible factor 1-alpha under hypoxic conditions to enhance cellular adaptation to inflammatory stress. Notably, this EV-mediated system can simultaneously suppress multiple inflammatory pathways, thereby creating a comprehensive immunomodulatory environment that cannot be achieved by conventional single-target therapies. Moving forward, the field must prioritize optimizing EV engineering strategies to enhance tissue-specific delivery and refine the spatiotemporal activation of the hypoxia-inducible factor 1-alpha/vascular endothelial growth factor axis. Such precision approaches will be important for maximizing therapeutic efficacy while minimizing potential complications associated with autoimmune disease interventions, ultimately paving the way for a new class of targeted nanotherapeutics.
